# Targeted-theranostic nanoparticles induce anti-tumor immune response in lung cancer

**DOI:** 10.1186/s12951-025-03542-4

**Published:** 2025-07-01

**Authors:** Zeinaf Muradova, Léna Carmès, Needa Brown, Fabien Rossetti, Romy Guthier, Sayeda Yasmin-Karim, Michael Lavelle, Toby Morris, Eder Jose Guidelli, Mileni Isikawa, Sandrine Dufort, Guillaume Bort, Olivier Tillement, François Lux, Ross Berbeco

**Affiliations:** 1 Department of Radiation Oncology, Dana-Farber Cancer Institute, Harvard Medical School, Boston, 02215 USA; 2 Department of Radiation Oncology, Brigham and Women’s Hospital, Harvard Medical School, Boston, 02115 USA; 3 Department of Materials Science and Engineering, University of Central Florida, Orlando, 32816 USA; 4 Department of Physics and Applied Physics, University of Massachusetts Lowell, Lowell, 01854 USA; 5 NH TherAguix SA, Meylan, 38240 France; 6 Université Claude Bernard Lyon 1, CNRS, Institut Lumière Matière, Villeurbanne, 69100 France; 7 Institut Curie, PSL Research University, CNRS, UMR9187, INSERM, U1196, Chemistry and Modeling for the Biology of Cancer, Orsay, F-91400 France; 8 Université Paris-Saclay, CNRS, UMR9187, INSERM, U1196, Chemistry and Modeling for the Biology of Cancer, Orsay, F-91400 France; 9 Institut Universitaire de France (IUF), Paris, 75005 France; 10 Departamento de Física-Faculdade de Filosofia, Ciências e Letras de Ribeirão Preto, Universidade de São Paulo, Ribeirão Preto, São Paulo, 14040- 901 Brazil

## Abstract

High atomic number metal-based nanoparticles (NPs) enhance radiotherapy efficacy by improving tumor multimodal imaging and intensifying radiation dosage. AGuIX-Bi represents a theranostic NP comprising gadolinium and bismuth chelates grafted on a polysiloxane core. We hypothesize that modulating the surface of AGuIX-Bi with targeted peptides will enhance the therapeutic responses of radiation therapy. The cyclic Arg-Gly-Asp (cRGD) peptide has a high affinity to RGD-binding integrins overexpressed on various tumor cells, including lung cancer. In the present study, we introduced cRGD peptides onto the surface of AGuIX-Bi NPs using a PEG spacer to form an amide bond with the free amino functions present at the surface of the NPs. In vitro, AGuIX-Bi-cRGD showed enhanced internalization of NPs via integrin binding and increased radiosensitization. In murine Lewis lung carcinoma (LLC) tumors, AGuIX-Bi-cRGD exhibited increased accumulation and retention in tumors without causing systemic toxicity. Combined with fractionated irradiation, AGuIX-Bi-cRGD converted a “cold” LLC tumor microenvironment (TME) into a “hot” one by inducing the overexpression of the immunogenic cell death marker HMGB1 and increasing the density of tumor-infiltrating CD3^+^ CD8^+^ cytotoxic T cells and thereby delaying tumor growth and improving mice survival. Therefore, these results suggest a potential strategy for using targeted AGuIX-Bi-cRGD NPs to sensitize lung tumors to radiation and immunotherapies.

**Supplementary Information:**

The online version contains supplementary material available at 10.1186/s12951-025-03542-4.

## Graphical abstract

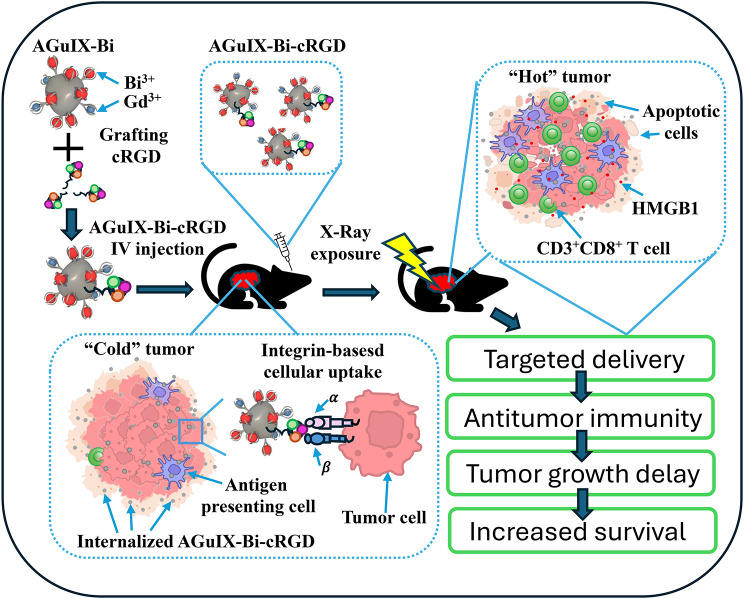

## Introduction

Lung cancer remains the leading contributor to cancer-related fatalities in men and women in the US [1, 2]. Non-small cell lung cancer (NSCLC) is the most frequent histological subtype of lung cancer, accounting for around 85% of all cases [3, 4]. Numerous studies [5–7] have shown that radiation therapy is an effective and well-tolerated noninvasive treatment option correlating with increased overall survival in NSCLC patients. But while radiation therapy is efficient, it is not a targeted antitumor treatment and frequently also damages healthy tissue [8–10]. Metal-based NPs (NPs) with a high atomic number (Z), such as bismuth (Z_Bi_ = 83) [11], gold (Z_Au_=79) [12], and gadolinium (Z_Gd_ = 64) [13], emit photo/Auger electrons when subjected to kilovoltage (kV) and megavoltage (MV) photon beams, thereby amplifying the radiation therapy dosage in treated tumor regions [14, 15]. Gadolinium-based compounds also act as T1 contrast agents, raising the possibility of magnetic resonance imaging (MRI)-guided radiation therapy [11, 16–18]. Due to the abnormal vasculature and the absence of lymphatic drainage in tumors, ultrasmall particles penetrate tumors primarily via the passive enhanced permeability and retention (EPR) effect and accumulate for long periods [19–21]. However, despite these benefits, the clinical translation of metallic NPs remains challenging due to poor targeting efficiency [22–24], heterogeneous tumor penetration [25, 26], rapid systemic clearance by phagocytes [27–29], and toxicity concerns [30, 31]. Overcoming these limitations necessitates the development of optimized nanoparticle designs that enhance tumor accumulation while minimizing off-target effects.

AGuIX^®^ (*A*ctivation and *G*uidance of *I*rradiation by *X*-ray) is a first generation of ultra small gadolinium-based NPs developed in 2011 [32]. AGuIX^®^ consists of a polysiloxane core on which gadolinium chelates are grafted resulting in a high concentration of gadolinium ions (∼ 15 gadolinium per NP), which serves a dual function - as a contrast agent for MRI as well as a local enhancer of radiation dosage [32, 33]. After extensive pre-clinical studies [34] that demonstrated the efficacy and safety of AGuIX^®^, several clinical trials were launched in combination with radiotherapy for brain metastasis (NCT02820454 [35, 36], NCT03818386, NCT04899908 [37]), glioblastoma (NCT04881032 [38]), cervical cancer (NCT03308604) [39, 40], and pancreatic and lung tumors (NCT04789486). The AGuIX-Bi is a second generation of gadolinium-based theranostic NPs produced by modifying the original AGuIX^®^ platform through a process involving the release of gadolinium ions in acidic conditions, followed by the addition of bismuth ions to achieve a molar ratio of 30Gd/70Bi per particle. Our pre-clinical studies in the NSCLC model demonstrated that adding higher Z bismuth ions in the AGuIX-Bi structure improved the therapeutic efficacy of these NPs while maintaining their MRI positive contrast properties [41]. Because of their versatile surface chemistry, NPs based on the AGuIX^®^ platform can be further functionalized with ligands that actively target tumors [42].

Of the numerous targeting ligands created so far, the cyclic tripeptide Arg-Gly-Asp (cRGD) stands out as an extensively researched tumor-targeting peptide [43–48]. The cRGD peptide has a high affinity towards several integrins that are overexpressed in NSCLC patients, including α_v_β_3_, α_v_β_5_, α_v_β_8_ [49, 50], and α_v_β_6_ [50, 51]. Metallic nanoparticles modified with RGD peptides offer advantages such as precise drug delivery [52–54], improved cellular uptake [48, 55, 56], and potential applications in non-invasive cancer imaging [57–60]. Furthermore, RGD-tagging assists in activating of an anti-cancer immune response through immunogenic cell death (ICD) mechanisms [61]. Previous studies have shown that radiation therapy [62, 63] and its combination with AGuIX^®^ NPs [64] triggers an accumulation of ICD markers, such as the high-mobility group protein 1 (HMGB1), which plays an essential role in dendritic cell (DC) maturation and antitumor specific T-cell responses [65–67].

**Scheme 1:**
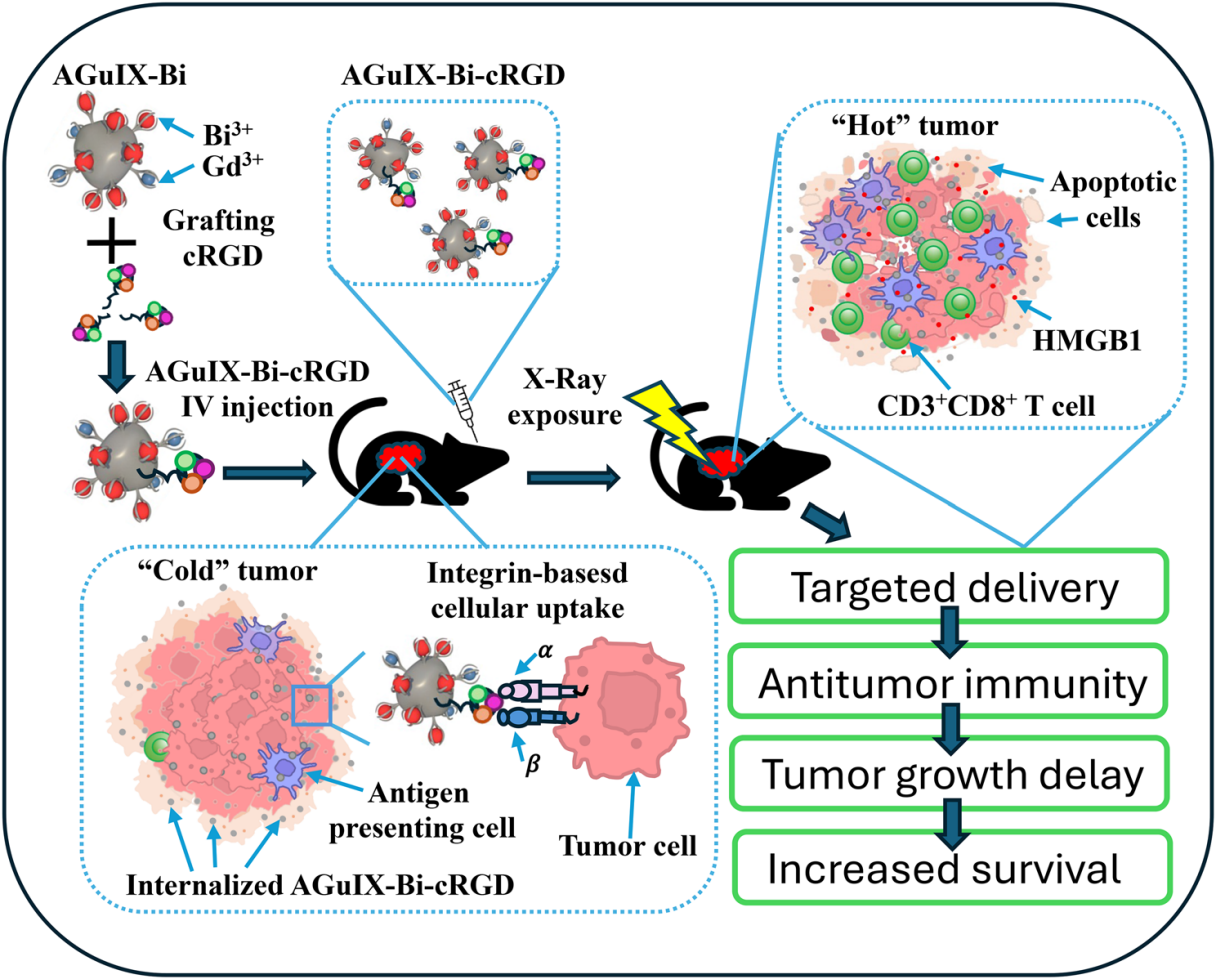
Schematic diagram of the synthesis, cellular uptake, and therapeutic action of AGuIX-Bi-cRGD nanoparticles, which transform immunologically “cold” tumors into “hot” ones when combined with radiation therapy by triggering the immunogenic cell death marker HMGB1 expression and CD3^+^ and CD8^+^ T cells infiltration

As illustrated in Scheme 1, here, we have grafted a cRGD peptide onto the surface of our previously described effective theranostic AGuIX-Bi NPs [41] (denoted further as AGuIX-Bi-cRGD) that improved their tumor targeting and immunotherapeutic capabilities. Specifically, we show that cRGD tagging enhances AGuIX-Bi NP internalization in both in vitro and in vivo models of lung cancer. Given their limited systemic toxicity, AGuIX-Bi-cRGD NPs improve the efficacy of radiation therapy and induce an antitumor immune response by modulating HMBG1 expression. Radiation-induced HMGB1 release from dying cells activates Toll-like receptor 4 (TLR4)-dependent DCs, which in turn mediate a cytotoxic T-lymphocyte (CTL) response against tumor cells [67]. Consistent with these observations, our results confirm that AGuIX-Bi-cRGD NPs boost radiation-induced CD3^+^ and CD8^+^ T cells infiltration in NSCLC tumors. Our findings indicate that targeted AGuIX-Bi-cRGD theranostic NPs could serve as a promising and clinically translatable approach to enhance the immune response initiated by radiation therapy.

## Experimental section

### Synthesis of AGuIX-Bi-Cy5.5-RGD

The AGuIX-Bi NPs were synthesized as per the protocol described by N. Brown et al. [41]. AGuIX-Bi NPs (3.16 w% Gd, 9.97w% Bi, 50 mg.mL^− 1^ NP final concentration in reaction, redispersed in MilliQ H_2_O) and Cy5.5-NHS ester (#2375105-86-3, Lumiprobe) were mixed in H_2_O (85.9 µM final concentration in reaction). The solution was stirred at RT for 30 min. The AGuIX-Bi-Cy5.5 was then purified by filtration using a vivaspin filter with a molecular weight cutoff (MWCO) of 5 kDa.

cRGD-PEG_2000_-NHS (#R-8891, Ruixibiotech) was added (250 mM, final concentration in reaction) to the AGuIX-Bi-Cy5.5 (at 100 mg.mL^− 1^ final concentration in reaction) in 10 mM PBS, and stirred for 2 h at RT. The NPs were purified by filtration using a vivaspin filter with a molecular weight cutoff (MWCO) of 5 kDa. Experimental details of assays characterizing nanoparticles physical and chemical characteristics are provided in Supplementary materials.

### Cell lines

Murine non-small cell lung cancer LL/2-Fluc-Neo/eGFP-Puro (LLC) cells (#CL073-STAN, Imanis) were cultured in DMEM-GlutaMax (#31966047, Life Technologies), supplemented with 10% heat-inactivated (HI) fetal bovine serum (FBS) (#10082147, Gibco), 100 IU/ml penicillin–streptomycin (#15140122, Life Technologies), 2 µg.mL^− 1^ of Puromycin (#A1113802, Thermo Fisher Scientific), and 1.25 mg.mL^− 1^ Geneticin (#10131027, Life Technologies). Human non-small cell lung cancer A549 cells (#CCL-185, ATCC, Fisher Scientific) were maintained in RPMI-1640 (#11835055, Gibco) media, supplemented with HI FBS (#10082147, Gibco, USA), and 100 IU.mL^− 1^ penicillin–streptomycin (#15140122, Life Technologies), and HUVEC endothelial cell (#CRL-1730, ATCC, Fisher Scientific) cultured Endothelial Cell Growth Medium (#C-22010, Sigma-Aldrich). All cells were maintained under 5% CO_2_ humidified atmosphere at 37 °C. Experimental details of in vitro assays are provided in Supplementary materials.

### Animals

C57BL/6JRccHsd wild type 6-week-old female mice were purchased from Envigo (#043), maintained in standard cages with free access to food and water, and were allowed to acclimate to the animal facility for at least 1 week before experimentation. For in vivo experiments, mice were inoculated subcutaneously into the dorsolateral right flank with 5 × 10^4^ LLC cells dispersed in 100 µL of base DMEM media and allowed to grow for 18–19 days. Mice bearing subcutaneous LLC tumors were randomized and given retro-orbital, i.v. injections of either 300 mg.kg^− 1^ of AGuIX-Bi or AGuIX-Bi-cRGD, or of saline. Seven hours post injection, tumors were exposed to local irradiation of 6 Gy, followed by 6 Gy fractions, given daily for the following 2 days.

### Irradiation

Small Animal Radiation Research Platform (SARRP, Xstrahl, Inc., Suwanee GA) was used to deliver radiation dose for in vivo and in vitro studies. For computed tomography (CT) image-guided radiation studies in vivo, mice were anesthetized with isoflurane vapor (1–3% isoflurane mixture and an airflow of 1.5 L min^− 1^) and CT images were taken at 65 kVP energies (0.8 mA current with a 1 mm Al filter). CT images were used to identify the tumor isocenter in MuriSlice and a single dose of 6 Gy was delivered to tumors three days in a row with a 1 × 1 cm^2^ collimated field using 220 kVp, 13 mA, and 0.15 mm copper (Cu) filter. For in vitro studies, various doses of irradiation were delivered to cells seeded in plates with an open field using 220 kVp, 13 mA, and 0.15 mm Cu filter.

### Biodistribution and pharmacokinetics

ICP-MS measurements were carried out to confirm and quantify the presence of Gd and Bi in various organs. Mice were injected i.v. retro-orbitally, with 300 mg.kg^− 1^ (∼ 6 mg per animal) of either AGuIX-Bi or AGuIX-Bi-cRGD, and were sacrificed 7 h, 24 h, 48 h, and 20 days later (4–8 animals per time point) post injection. Tumors, blood, and vital organs such as the liver, kidney, spleen, lungs, and heart were collected for ICP-MS analysis. Collected samples were dissected, weighed, and digested in 69% HNO_3_ using a Multiwave 5000 microwave (Anton Paar, Austria), and analyzed using ICP-MS Nexion 2000 B (Perkinelmer).

### In vivo antitumor efficacy

The mice were given the identical treatment as described in section Animals. The tumor volume and body weight were recorder every 48 h. Tumor dimensions were measured with caliper. Tumor volume (TV), and relative tumor volume (RTV) were calculated using the following formulas: $\:TV=0.5\:\text{x}\:length\:\text{x}\:{width}^{2}$, and $\:RTV=\left(TV\:on\:measured\:day\right)\:/\:\left(TV\:on\:day\:0\right)$.

### In vivo toxicology and immunohistochemistry

Mice were treated as described in section Animals. After 24 h post-treatments, blood, tumors and other vital organs (liver, lungs, spleen, kidney, and heart) were collected for toxicology assessment. A complete blood count (CBC) was recorded on freshly collected blood at the DFCI Animal Research Facility. Serum was extracted from clotted blood and analyzed for blood urea nitrogen (BUN), and Gamma-glutamyl transferase (GGT) levels at the Beth Israel Deaconess Medical Preclinical Murine Pharma Core. Organs were fixed with 10% NBF (# 4499–10 L, Medsupply Partners) for 48 h, embedded in paraffin, and sections were labelled with hematoxylin and eosin at Brigham and Women’s Hospital Pathology Core. Toxicity levels in organs were accessed by a pathologist at the Harvard Medical School Rodent Histopathology Core.

Tumor sections were further stained for CD3 and CD8. Sequential immunofluorescent staining was performed on the Leica Bond III automated staining platform with use of the Leica Biosystems Refine Detection Kit, at Brigham and Women’s Hospital Pathology Core. Briefly, anti-CD8 antibody (#98941, Cell Signaling Technology) was used at a 1:200 dilution, utilizing a citrate antigen retrieval (#AR9961, Leica Biosystems) for 30 min and visualized with Alexa Fluor 555. Following stripping with EDTA (#AR9640, Cell Signaling Technology), samples were further stained with anti-CD3 antibody (#99940, Cell Signaling Technology) at 1:150 dilution, visualized with Alexa Fluor 647, and counterstained with DAPI. For HMGB1 analysis, after the deparaffinization and antigen retrieval (in 10 mM sodium citrate buffer, pH 6 at 97 °C for 20 min) biopsy sections from mice were blocked for 1 h in 20% FBS in PBS and incubated overnight at 4 °C with the anti-HMGB1 antibody (#3935S, Cell Signaling Technology) at a 1:50 dilution. Samples were further incubated for 2 h with Alexa Fluor 546-conjugated goat anti-rabbit (#A-11012, Invitrogen) at a 1:500 dilution plus DAPI. All images were acquired on a fluorescence microscope (Axio Observer 7, Carl Zeiss Microscopy) using a 100X oil objective; 30 images per each condition were analyzed using an ImageJ2 (2.14.0/1.54f) Triangle Auto Threshold method.

### Statistical analysis

**Fig. 1:**
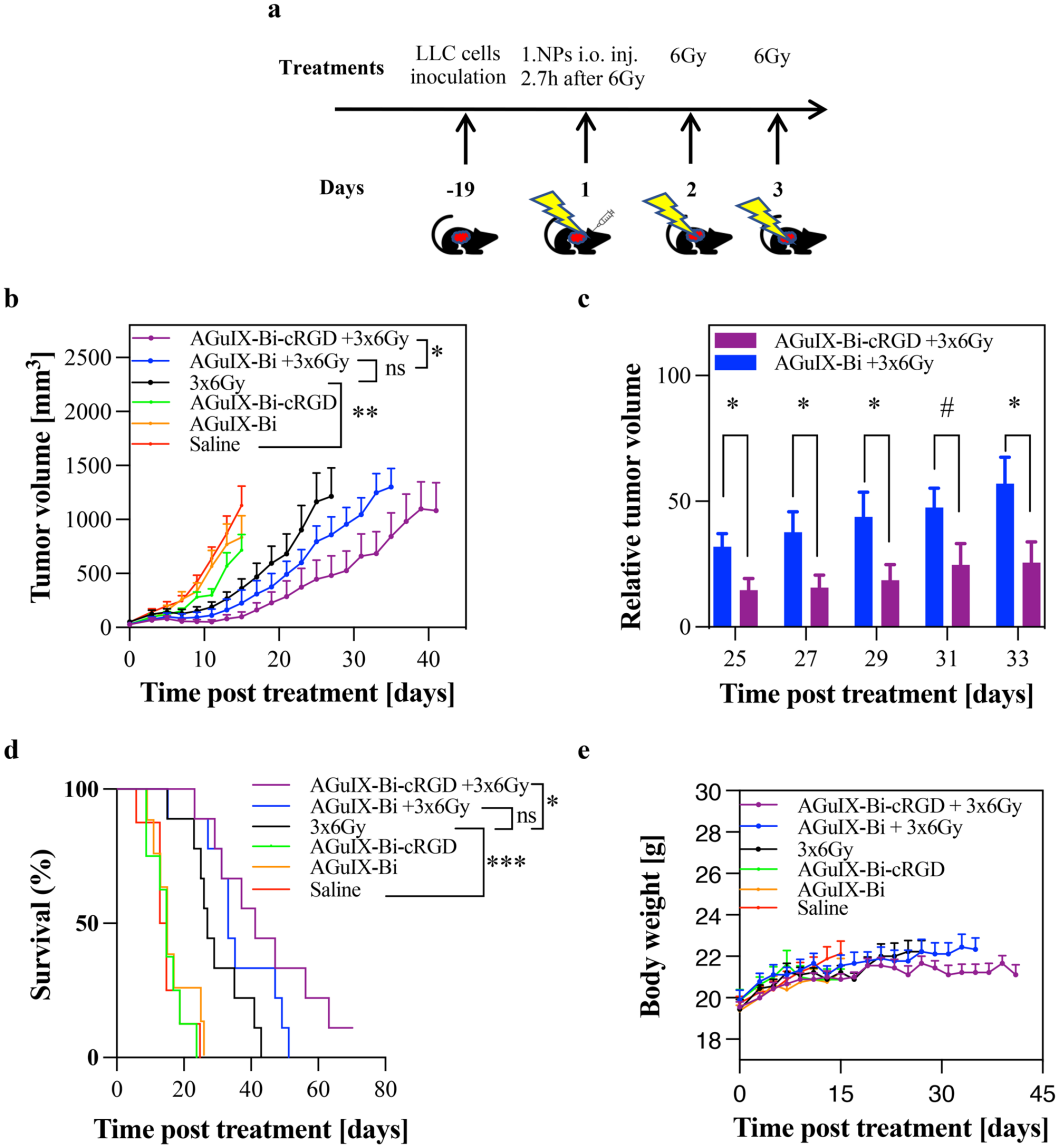
Use of AGuIX-Bi-cRGD plus radiation therapy enhances antitumor efficacy in vivo (**a**) Scheme of the experimental procedures (**b**) In vivo tumor volume (**c**) Relative tumor volume (**d**) Kaplan-Meier analysis of survival and (**e**) Changes in body weight in LLC tumor-bearing mice (*n* = 8 in non-irradiated and *n* = 9 in irradiated groups. ***p < 0.001, **p < 0.01, *p < 0.05, #*p* = 0.07, ns = not significant, two-tailed unpaired Student’s t test. Graphs show mean and error ± SEM)

Statistical analysis was performed with GraphPad Prism Version 10.2.3 for Mac (GraphPad Software, Boston) using two-tailed Student’s t-test. The significance levels depicted in the figure graphs are *P < 0.05, **P < 0.01, ***P < 0.001, and ****P < 0.0001. Details regarding the sample size (n), statistical tests, and adjustments for data representations are provided in the respective figure legends. For Fig. 1b, c and e, to mitigate the missing data points, the last observation carried forward method [68, 69] was implemented until 5 mice per group has dropped out.

## Results and discussion 

### In vivo antitumor efficacy

The therapeutic efficacy of targeted AGuIX-Bi-cRGD and AGuIX-Bi in combination with fractionated X-ray irradiation was evaluated in vivo. Six groups of LLC subcutaneous tumor-bearing mice were intravenously treated with 300 mg.kg− 1 of AGuIX-Bi, AGuIX-Bi-cRGD, or PBS, followed by daily X-ray irradiation at a dose of 6 Gy/fraction (220 kVp, 13 mA, 0.15 mm Cu filter) for a total of 3 fractions on consecutive days (Fig. 1a, Fig S1). As shown in Fig. 1b, targeted AGuIX-Bi-cRGD combined with 3 × 6 Gy not only delayed tumor growth, relative to the 3 × 6 Gy group (p < 0.05), but also yielded complete regression of tumor in treated mice (Fig S2). The relative tumor volume (RTV) indicated that the combination treatment of AGuIX-Bi-cRGD + 3 × 6 Gy significantly (p < 0.05) reduced relative tumor growth over time compared to the AGuIX-Bi + 3 × 6 Gy group (Fig. 1c). Figure 1d also shows that the group of mice treated with the combination AGuIX-Bi-cRGD and 3 × 6 Gy exhibited a more prolonged median survival (p < 0.05) compared to mice receiving 3 × 6 Gy alone. In contrast, the combination of non-targeted AGuIX-Bi and 3 × 6 Gy showed no significant impact on tumor burden (Fig. 1b) or survival (Fig. 1d) compared to the 3 × 6 Gy group. While fractionated irradiation alone significantly inhibited tumor growth (p < 0.01) and improved survival (p < 0.001) compared with the saline group, complete regression was not observed in mice treated with 3 × 6 Gy or AGuIX-Bi + 3 × 6 Gy. Body weights of treated mice stayed stable across all treatments (Fig. 1e), indicating the absence of systemic toxicity. These results support the potent radio enhancing efficiency of the targeted AGuIX-Bi-cRGD NPs and their biosafety.

### Synthesis and characterization of AGuIX-Bi-cRGD

**Fig. 2:**
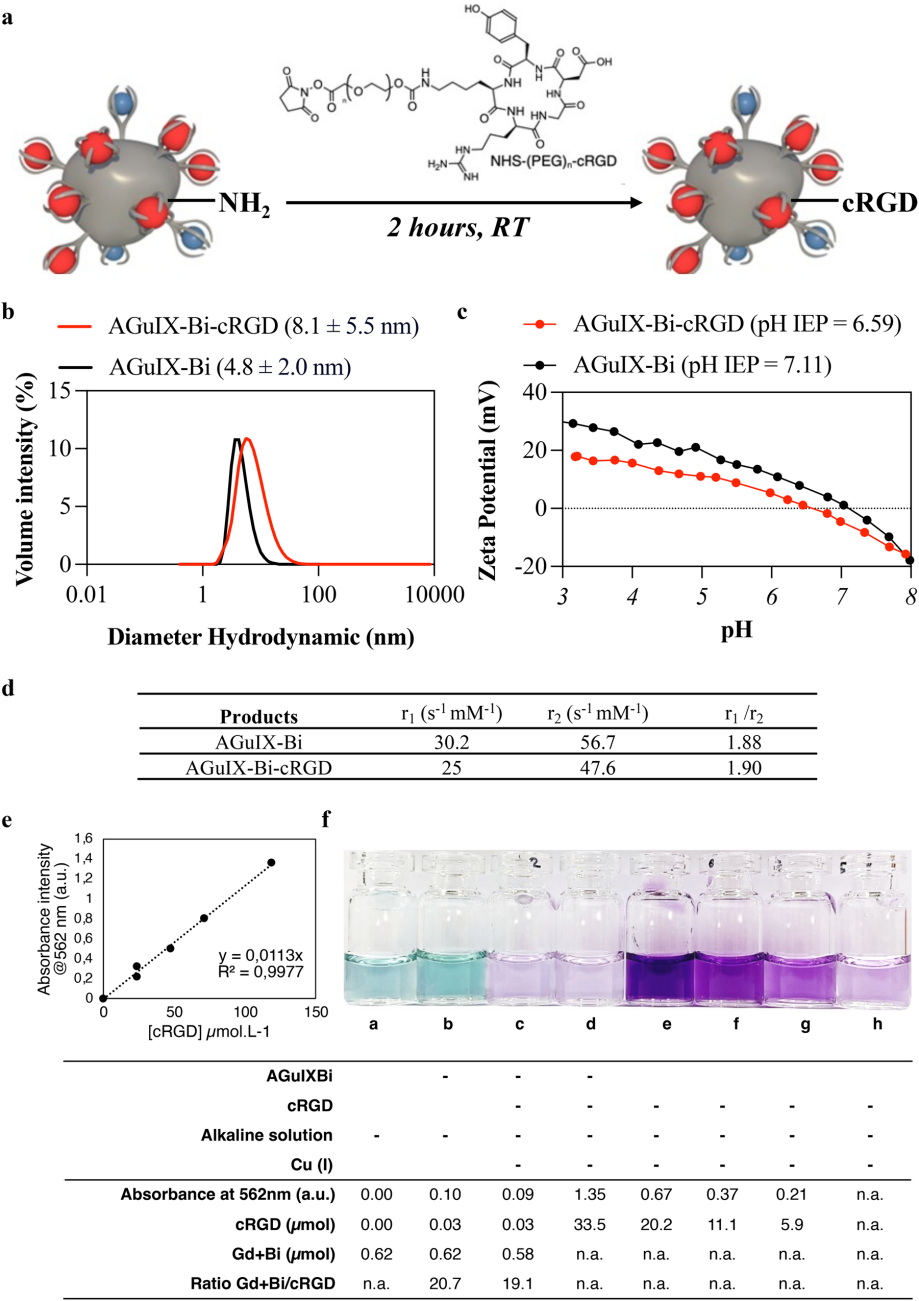
Synthesis and characterization of AGuIX-Bi-cRGD. (**a**) Synthesis scheme for functionalizing AGuIX-Bi with a cRGD peptide by amidation. (**b**) Measurement of hydrodynamic diameter. (**c**) ζ potential profiles. (**d**) Summary table comparing the physical characteristics of AGuIX-Bi and AGuIX-Bi-cRGD. Longitudinal relaxivity (r1) and transverse relaxivity (r2) are calculated based on gadolinium concentration (in mM). (**e**) Calibration curve for cRGD, quantified by BCA assay (**f**) Summary table of raw absorbances obtained with the BCA protein assay and molar quantities resulting from the calibration curve: samples were quantified spectroscopically on the following: (**a**) reference reagent solution, (**b**) AGuIX-Bi, (**c**) AGuIX-Bi + cRGD (unconjugated), (**d**) AGuIX-Bi-cRGD (conjugated), (**e**) Wash 1, (**f**) Wash 2, (**g**) Wash 3, and (**h**) Wash 4

AGuIX-Bi was synthesized from AGuIX^®^ NPs [41] using a three-step protocol. AGuIX^®^ NPs were placed in acidic conditions, which released some of the gadolinium ions and then purified by tangential filtration. Bi^3+^ was added to the NPs to obtain a Bi/Gd molar ratio of 70/30. cRGD attached to a PEG spacer was incorporated into the NPs through an amide bond, which formed because of a reaction between NHS ester and the free amino groups on the surface of the AGuIX-Bi NPs (Fig. 2a). After grafting, DLS measurements showed an increase in the hydrodynamic diameter (*D*_H_) from 4.8 ± 2.0 nm to 8.1 ± 5.5 nm (Fig. 2b), and a slow decrease in the isoelectric point from 7.11 to 6.59 due mainly to the reaction on the accessible amino functions (Fig. 2c). A comparable rise in the average D_H_ was noted in AGuIX^®^ NPs (D_H_ = 3.0 ± 1.1 nm) after functionalization with nanobodies AGuIX@A12 (D_H_ = 5.4 ± 3.1 nm), which confirms the modification of the particle surface [42]. Relaxivity measurements r_1_ and r_2_ of AGuIX-Bi-cRGD remained at high values after grafting (25 and 47.6 s^− 1^ mM^− 1^, respectively, at 1.4 T) with a r_1_/r_2_ ratio close to AGuIX-Bi (Fig. 2d). Our previous studies demonstrated that AGuIX-Bi particles, with a composition of 29.6% Gd and 70.4% Bi per particle, retained an MRI signal intensity comparable to AGuIX across several clinical scanners [41], reinforcing the potential of AGuIX-Bi-cRGD as a highly effective positive MRI contrast agent. Three methods were used to quantify cRGD on AGuIX-Bi-cRGD NPs: (i) indirect quantification in wash water, (ii) direct quantification by fluorescence titration, and (iii) BCA assay leading to respectively 16 and 17 metal ions (gadolinium + bismuth) per cRGD corresponding to about one cRGD per NP [70]. Direct titration by BCA assay was also performed, which led to a slightly different ratio of 19 metal ions per cRGD (Fig. 2e, f).

### In vitro internalization, radiosensitization, and toxicity

**Fig. 3:**
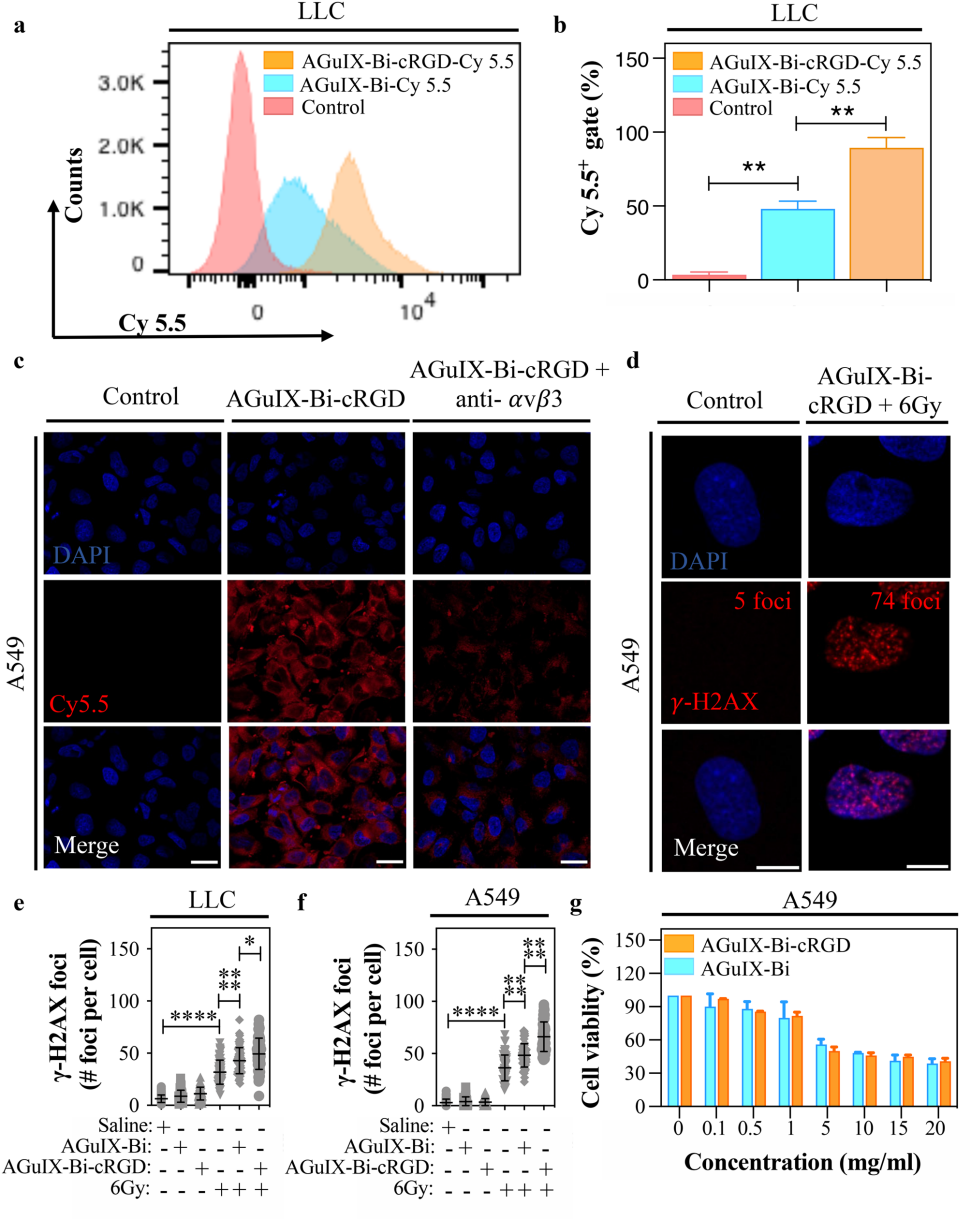
Internalization, radiosensitization, and toxicity of AGuIX-Bi and AGuIX-Bi-cRGD NPs in vitro (**a**) Flow cytometric histogram overlays of LLC cells exposed for 24 h to Cy 5.5-labeled AGuIX-Bi or to AGuIX-Bi-cRGD NPs. (**b**) Quantitative analysis of the fluorescence intensity of Cy 5.5-labeled AGuIX-Bi or AGuIX-Bi-cRGD NPs in LLC cells. (**c**) Representative images of A549 cells treated for 2 h with anti-αvβ3 integrin antibodies, prior to exposure to Cy 5.5-labeled AGuIX-Bi-cRGD NPs. Scale bar: 20 μm. (**d**) Representative images of γ-H2AX foci numbers in A549 cells exposed to 1 mg.mL^− 1^ of AGuIX-Bi-cRGD NPs in combination with 6 Gy. Scale bar: 10 μm. (**e**) Quantification of γ-H2AX foci numbers in LLC and (**f**) A549 cells exposed to 1 mg.mL^− 1^ of AGuIX-Bi or AGuIX-Bi-cRGD NPs alone or in combination with 6 Gy. (**g**) Cytotoxicity of various concentrations (0.1–20 mg.mL^− 1^) of AGuIX-Bi or AGuIX-Bi-cRGD NPs measured by the MTT assay on A549 cells. (*n* = 3 in each group, ****p < 0.0001, **p < 0.01, *p < 0.05, two-tailed unpaired Student’s t-test. Graphs show mean ± SD)

Cellular uptake for Cy 5.5 labeled AGuIX-Bi and AGuIX-Bi-cRGD NPs was studied in vitro. For flow cytometry analysis, LLC cells expressing GFP were treated with 1 mg.mL^-1^ of NPs for 24 h (Fig. 3a, b). Initial gating for GFP expression (Fig S3 a) was used to eliminate dead cells, followed by gating for Cy 5.5 + and Cy 5.5- cell populations (Fig S3 b). As shown in Fig. 3a, LLC cells with targeted AGuIX-Bi-cRGD NPs at 37 °C had nearly 2 times more the fluorescence intensity of those with non-targeted AGuIX-Bi, with mean values (Fig. 3b) of 89.43% and 48.03%, respectively. Additional receptor-blocking experiments were conducted to further validate the role of integrin-mediated endocytosis in AGuIX-Bi-cRGD NPs uptake. RGD-binding integrins are heterodimeric cell surface receptors that specifically recognize the RGD motif in their ligands [71, 72]. The cyclic RGD peptide is primarily known for its selective binding to α_v_β_3_ integrin [73–75]. To assess this interaction, A549 cells were pre-incubated with anti-integrin α_v_β_3_ antibody for 2 h prior to exposure to Cy 5.5 labeled AGuIX-Bi-cRGD NPs. Blocking these integrins led to a significant but incomplete reduction in Cy5.5 fluorescence intensity within A549 cells (Fig. 3c), indicating decreased nanoparticle internalization. Beyond α_v_β_3_, cyclic RGD peptides also bind to α_v_β_5_ [76, 77], α_IIb_β_3_ [78], and α_5_β_1_ [79]. In the A549 lung cancer cell line, integrin β5 [80] and α5 [81, 82] are highly overexpressed. Blocking these integrins with anti-α5 and β5 antibodies led to a greater reduction in Cy 5.5-labeled AGuIX-Bi-cRGD NPs uptake (Fig S4). These findings confirm that cRGD peptides on NPs retain biological activity, targeting integrins and enabling receptor-based endocytosis.

The γ-H2AX analysis, a widely recognized indicator for DNA double-strand breaks (DSBs) [83–85], was performed to evaluate whether in vitro cellular uptake rate of AGuIX-Bi or AGuIX-Bi-cRGD NPs is correlated with an increased sensitivity to irradiation in A549 and LLC cells. The cells were treated with 1 mg.mL^− 1^ of AGuIX-Bi or AGuIX-Bi-cRGD NPs, followed by a single dose of 6 Gy irradiation. After 30 min of irradiation, the number of γ-H2AX foci per cell was determined (Fig. 3d), as described [41]. Both types of lung cancer cells exhibited a similar response to the combination treatments, showing a significantly higher number of foci per nucleus in cells treated with AGuIX-Bi or AGuIX-Bi-cRGD NPs along with irradiation compared to those exposed to irradiation alone (Fig. 3e, f). The combination of irradiation and AGuIX-Bi-cRGD significantly enhanced the average number of γ-H2AX foci per cell (*p* < 0.05 in LLC and *p* < 0.0001 in A549 cells) compared to the number in cells treated with non-targeted AGuIX-Bi NPs plus irradiation cells, revealing that targeted AGuIX-Bi-cRGD NPs can increase the effects of irradiation by accumulating DNA double-strand breaks in lung cancer cells.

To further evaluate the in vitro cytotoxicity effects of AGuIX-Bi and AGuIX-Bi-cRGD NPs, we used the MTT assay on A549 cells. After 4 h of incubation with AGuIX-Bi or AGuIX-Bi-cRGD NPs at doses ranging from 0.1 mg.mL^− 1^ to 20 mg. mL^− 1^, the viability of treated A549 cells decreased with increasing concentration of both NPs (Fig. 3g). No significant differences in cytotoxicity were observed between AGuIX-Bi and AGuIX-Bi-cRGD NPs in the absence of irradiation. Similarly, AGuIX-Bi-cRGD NPs showed minimal toxicity in HUVEC endothelial cells (Fig S5). These results demonstrate that grafting the cRGD peptide on the surface of AGuIX-Bi NP’s surface does not decrease cell viability without irradiation.

### In vivo biodistribution

**Fig. 4:**
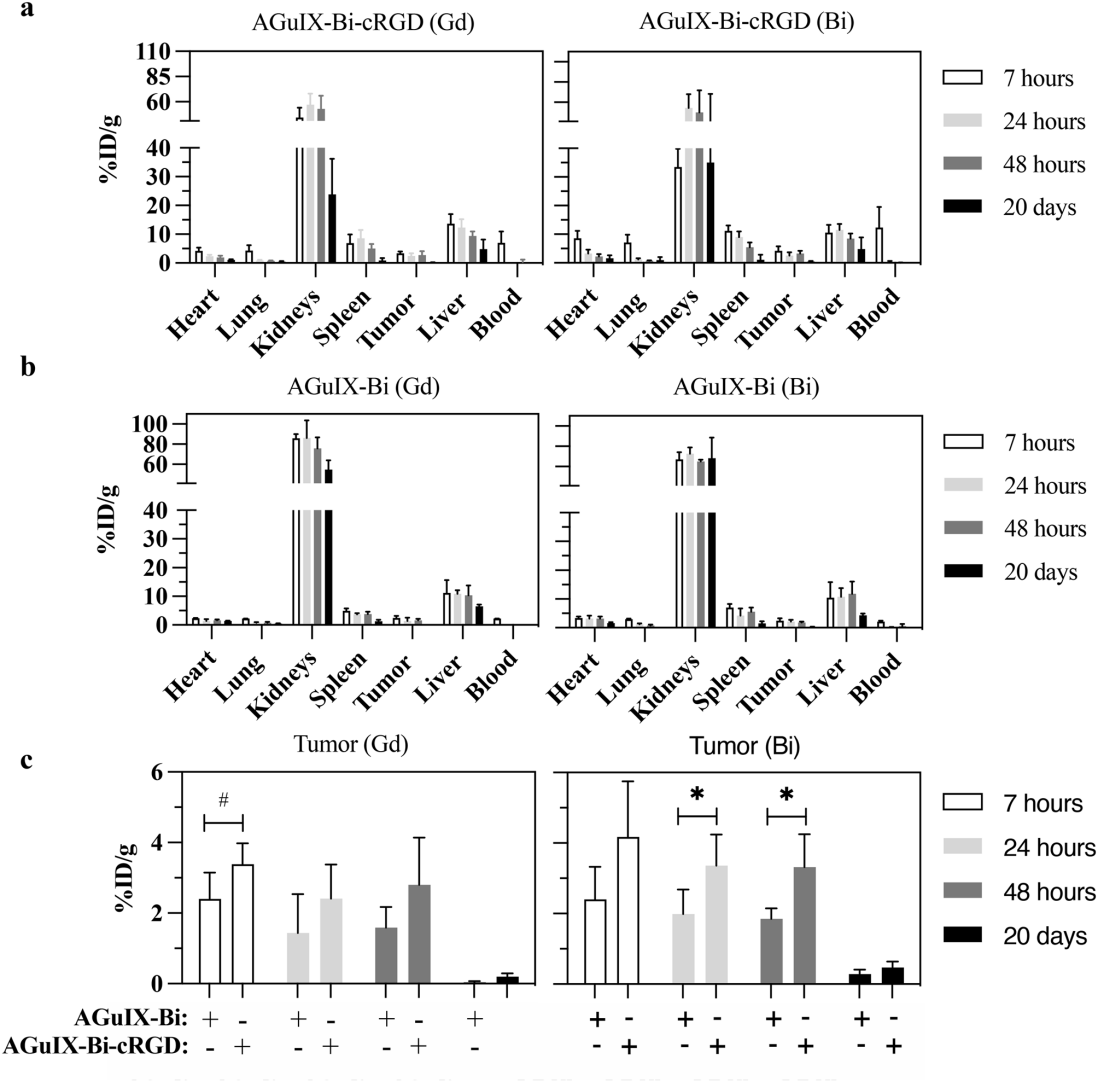
ICP-MS biodistribution analysis of AGuIX-Bi and AGuIX-Bi-cRGD in vivo. Comparison of biodistribution in various organs based on gadolinium or bismuth quantification of (**a**) AGuIX-Bi-cRGD or (**b**) AGuIX-Bi. (**c**) Intratumoral gadolinium or bismuth levels at different time points. Data are presented as percentage of injected dose per gram of tissue (%ID.g^− 1^) (*n* = 4 or 8 in each group. *p < 0.05, #*p* = 0.084 two-tailed unpaired Student’s t-test. Graphs show mean ± SD)

We next assessed the biodistribution of AGuIX-Bi-cRGD relative to AGuIX-Bi, after retro-orbital administration of NPs at various time points in mice bearing lung subcutaneous tumors. The mice were sacrificed at different time points (7 h, 24 h, 48 h, and 20 days) after administration of NPs, the organs of interest were digested, and gadolinium and bismuth ions were quantified by inductively coupled plasma mass spectrometer (ICP-MS). Both NPs displayed similar biodistribution patterns with renal elimination, limited uptake in the liver (Fig. 4a, b), and no significant accumulation in other organs over time (Fig S6 a-f ). For tumor uptake, a significantly higher increase (*p* < 0.1) was observed for AGuIX-Bi-cRGD at the different time points (Fig. 4c), indicating longer retention for peptide-functionalized NPs. Both AGuIX-Bi and AGuIX-Bi-cRGD NPs continued to be detected in tumors even 20 days after systemic administration. All quantifications were done on gadolinium and bismuth, and the Bi/Gd ratio (Fig S6 g) remained relatively stable in the different organs at different times, pointing to in vivo stability of the AGuIX-Bi-cRGD.

### In vivo pharmacological toxicity

**Fig. 5:**
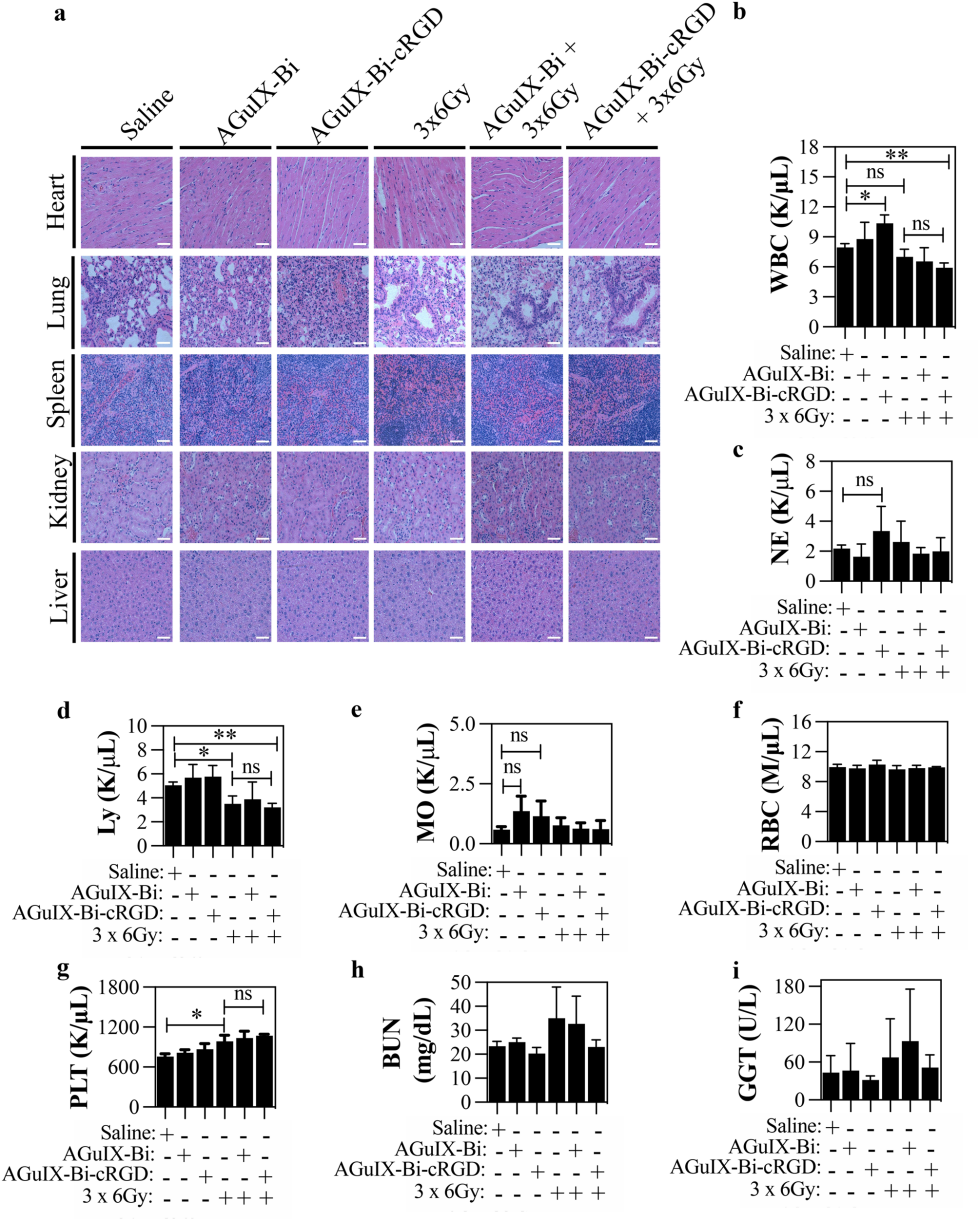
Evaluation in vivo toxicity of AGuIX-Bi and AGuIX-Bi-cRGD in combination with RT (**a**) Representative images of H&E-stained tissue slices of major organs (heart, lung, spleen, kidney, liver). Scale bar: 50 μm. (**b**) Total white blood cell (WBC) counts (**c**) Neutrophil (NE) (**d**) Lymphocyte (Ly) (**e**) Monocyte (Mo) (**f**) Red blood cell (RBC) and (**g**) Platelet (PLT) counts (**h**) Serum blood urea nitrogen (BUN) and (**e**) Gamma-glutamyl transferase (GGT) levels (*n* = 2 or 3 in each group. **p < 0.01, *p < 0.05, ns = not significant, two-tailed unpaired Student’s t-test. Graphs show mean ± SD)

We also used histological and hematological analyses to evaluate the in vivo toxicity of AGuIX-Bi and AGuIX-Bi-cRGD NPs plus fractionated irradiation in C57BL/6 mice bearing LLC tumors after 24 h of treatment. Histopathological changes in the main organs, including the heart, lung, spleen, lung, kidney, and liver, were assessed with H&E staining and recorded microscopically (Fig. 5a): no apparent toxicity-related lesions were observed in the major organs. Complete blood count sampling in the 3 × 6 Gy, AGuIX-Bi + 3 × 6 Gy, and AGuIX-Bi-cRGD + 3 × 6 Gy groups (Fig. 5b-g and Fig S7 a-i ) revealed no significant increases in white blood cells (Fig. 5b-e and Fig S7 a-b), red blood cells (Fig. 5f and Fig S7 c-f), and platelets (Fig. 5g and Fig S7 g-i). However, compared to untreated control mice, the white blood count was significantly increased (*p* < 0.05) after treatment with AGuIX-Bi-cRGD and was reduced after combination with fractionated irradiation (*p* < 0.01) (Fig. 5b). A similar increase in white blood cells has been reported after systemic treatment with gold NPs [86, 87], suggesting that metal-based NPs affect the body’s immune system. As shown in Fig 5d,.  3×6 Gy alone (*p* < 0.05) or in combination with AGuIX-Bi-cRGD (*p* < 0.01) also decreased the number of lymphocytes. Hematologic toxicity is common among the immediate consequences of radiation therapy [88–91], however, the count of white blood cell counts typically show a gradual increase over time and eventually stabilize within the normal range [92, 93]. We found that 3 × 6 Gy alone increased a number of platelets (*p* < 0.05) and platelet crit (*p* < 0.05) (Fig. 5g and Fig S7 g, respectively). Results of the serum biochemistry study showed no significant differences (*p* > 0.05) in levels of blood urea nitrogen (Fig. 5h) and of gamma-glutamyl transferase (Fig. 5i) relative to those in non-treated controls. Together, the above results indicate that AGuIX-Bi-cRGD and AGuIX-Bi NPs, when combined with fractionated irradiation, have minimal systemic toxicity in vivo.

### Evaluation of immunogenicity

**Fig. 6:**
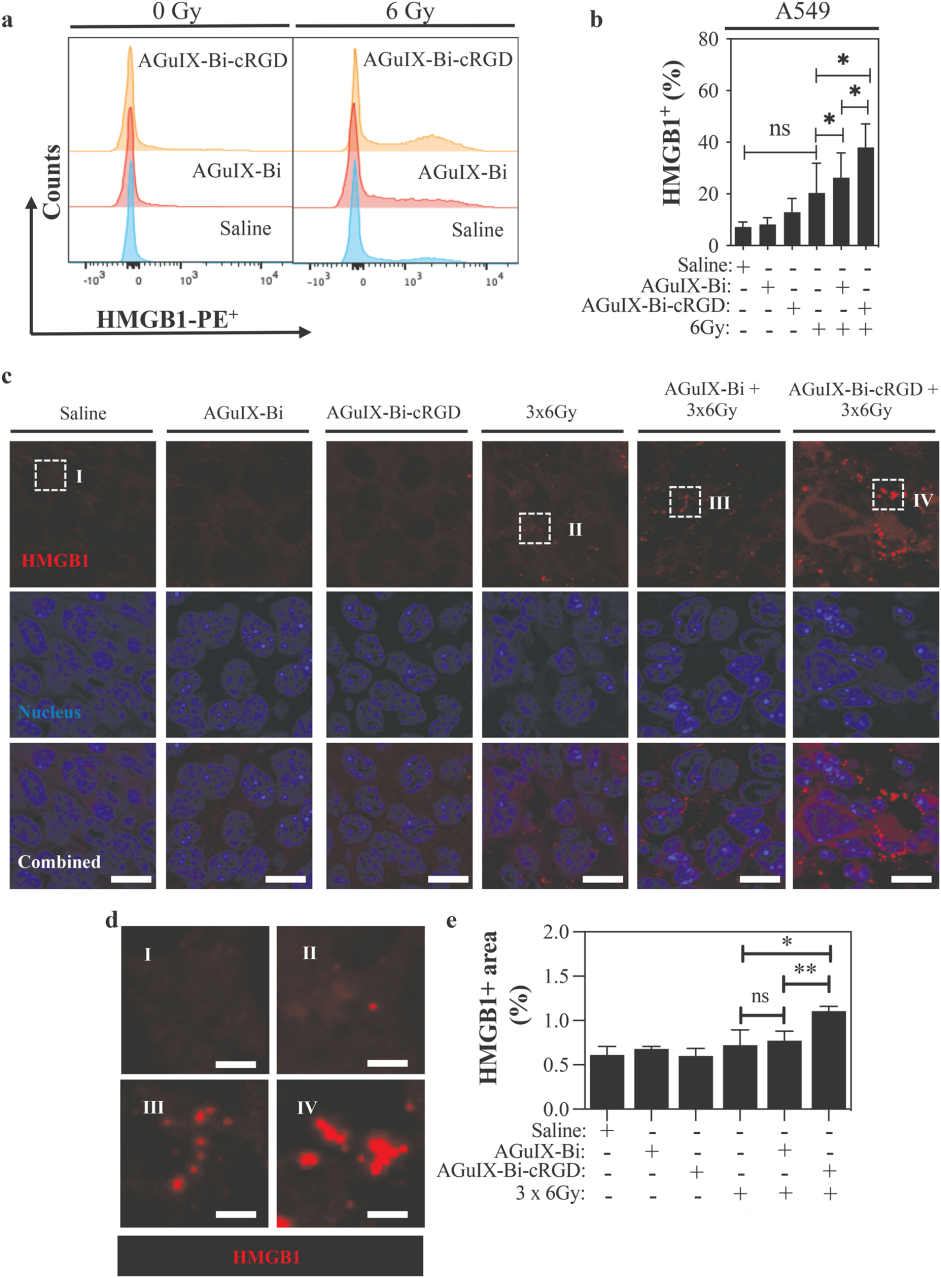
Use of AGuIX-Bi-cRGD plus irradiation induces the expression of HMGB1 in in vitro and in vivo models of lung cancer (**a**) Flow cytometry histogram overlays and (**b**) Quantitative analysis of the fluorescence intensity of A549 cells expressing HMGB1, following treatment with 1 mg.mL^− 1^ of AGuIX-Bi or AGuIX-Bi-cRGD NPs alone, or in combination with 6 Gy (*n* = 4) (**c**) Representative immunofluorescence (IF) images showing HMGB1 expression in tumor tissues. Scale bar: 20 μm; (**d**) Higher magnification details of HMGB1 expression in I, II, III and IV regions are shown. Scale bar: 5 μm (**e**) HMGB1 positive area quantification, 30 images per condition from different parts of the tumor were acquired and expression-positive areas were quantified using by Image J. (*n* = 3 in each group. *p < 0.05, **p < 0.01, ns = not significant, Student’s t-test. Graphs show means ± SD)

To explore the activation of immunogenic cell death markers, we employed flow cytometry, ELISA, and an immunofluorescence assay to measure both the cellular expression and extracellular release of HMGB1 protein in A549 cells and LLC subcutaneous tumor biopsies. A549 cells were treated for 4 h with 1 mg.mL^− 1^ of AGuIX-Bi or AGuIX-Bi-cRGD NPs, the nanoparticle suspensions were replaced with a complete growth media, and cells exposed to a single irradiation dose of 6 Gy. Cellular HMGB1 expression was determined by flow cytometry 24 h post-irradiation (Fig S8). As shown in Fig. 6a, compared to the control groups, irradiation alone and or in combination with each of the two types of NPs enhanced the expression of HMGB1 protein. However, the combination of radiation treatment and AGuIX-Bi-cRGD (Fig. 6b) led to a substantial synergistic cytotoxic effect and significantly increased HMGB1 expression compared to irradiation alone (*p* < 0.05) or to AGuIX-Bi plus irradiation (*p* < 0.05). At the same time, we analyzed the amount of HMGB1 released in A549 cell culture medium by ELISA. Our results showed that the combination of targeted AGuIX-Bi-cRGD and irradiation significantly increased HMGB1 release into the medium 96 h post-treatment compared to irradiation alone (*p* < 0.05) or irradiation combined with non-targeted AGuIX-Bi (Fig S9).

We next investigated whether targeted AGuIX-Bi-cRGD and AGuIX-Bi plus fractionated irradiation could enhance the HMGB1 expression in vivo. C57BL/6 mice bearing LLC tumors were treated as described (Fig. 5a). Twenty-four hours post-treatment, tumors were formalin-fixed, and paraffin-embedded sections through the tumor were stained for HMGB1. Using ex vivo florescence microscopy images (Fig. 6c and d), we quantified the fluorescence intensity in 30 images acquired from different regions of the tumor per condition. Consistent with the in vitro findings (Fig. 6b), a significant increase in fluorescence signal was observed in the AGuIX-Bi-cRGD + 3 × 6 Gy group compared to the 3 × 6 Gy (*p* < 0.05) and AGuIX-Bi + 3 × 6 Gy (*p* < 0.01) groups (Fig. 6e).

Several studies report that increased HMGB1 expression in the TME increases lymphocyte infiltration [94, 95]. Metallic nanoparticles can also modulate the TME, regulating dendritic cells [96–98], macrophages [99–101], T cells [102, 103], and NK cells [104, 105] to boost antitumor immunity. For instance, RGD-targeted ferrite nanoparticles (RGDMnFe2O4) activate the STING pathway in dendritic cells, enhancing CD8^+^ and CD4^+^ T cell infiltration and activation [97]. Similarly, RGD-functionalized quantum dots promote the expression of ICD markers, DC maturation, and tumor suppression [106].

**Fig. 7:**
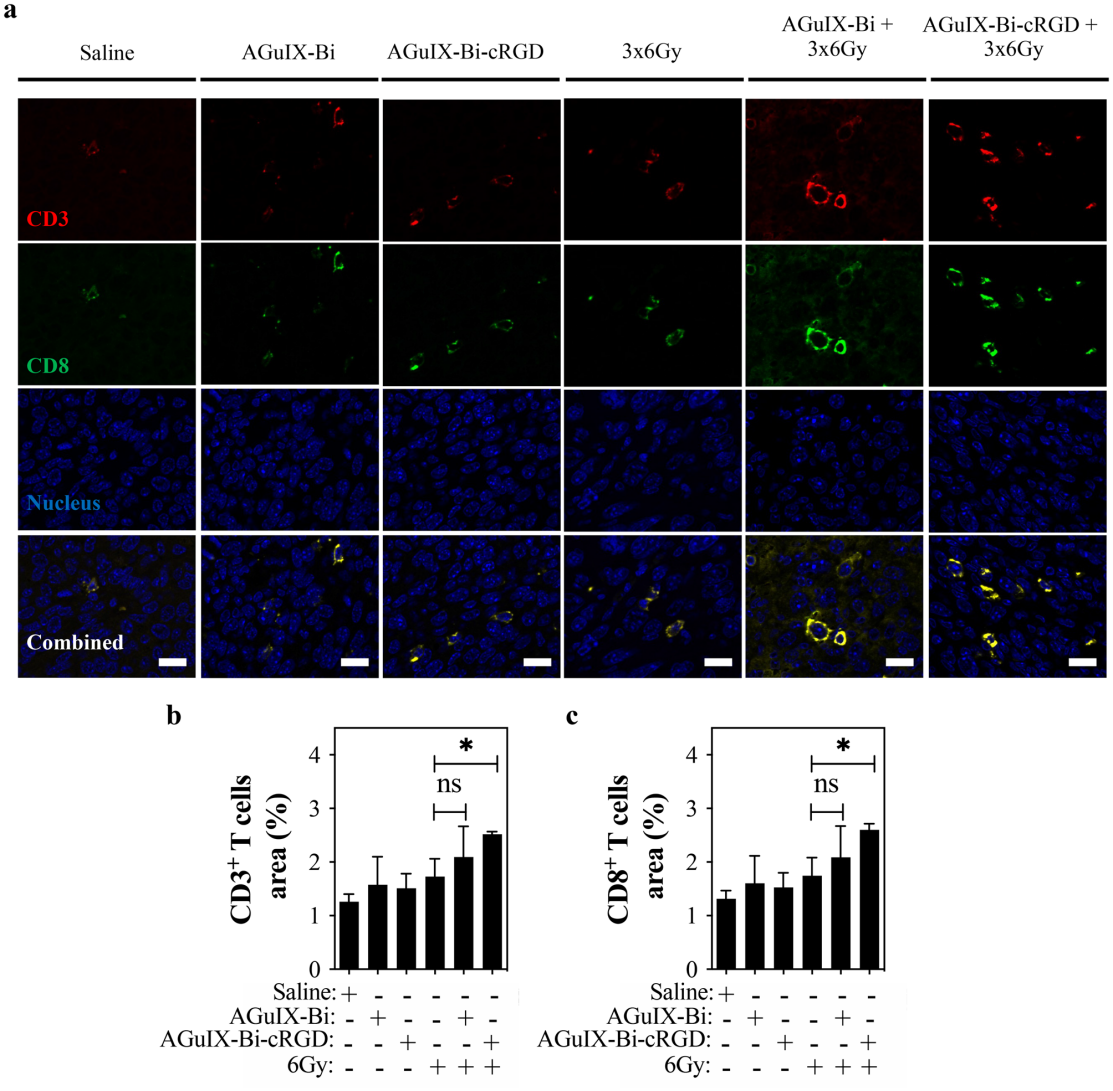
Combination of AGuIX-Bi-cRGD and irradiation promotes infiltration of cytotoxic T lymphocytes in the tumor microenvironment (**a**) Representative immunofluorescence (IF) images of CD3- and CD8-stained tumor tissues. Scale bar: 20 μm; (**b**) CD3^+^ and (**c**) CD8^+^ T cell area quantification, 30 images per condition from different parts of the tumor were acquired, and expression positive areas were quantified by Image J. (*n* = 3 in each group. *p < 0.05, ns = not significant, two-tailed unpaired Student’s t-test. Graphs show means ± SD)

Among the most potent antitumor lymphocytes, a high accumulation of CD8^+^ T cells in tumors is associated with a favorable prognosis in lung cancer [107, 108]. Furthermore, extensive research on the role of CD8^+^ T cells in irradiation-induced anti-tumor responses has shown that depletion of these cells significantly abolishes the therapeutic efficacy of radiation therapy [109–111] and prevents the occurrence of the abscopal effect, a phenomenon where irradiation at a localized site triggers systemic antitumor immunity [112–115]. These findings highlight CD8^+^ T cells’ essential role in enhanced antitumor effects. To examine lymphocyte infiltration, we stained paraffin-embedded sections from the same tumor sections used for HMGB1 analysis for CD3 and CD8 receptors (Fig. 7a). Quantification of the intensity in fluorescence microscopy images showed that, in comparison to 3 × 6 Gy and AGuIX-Bi plus 3 × 6 Gy groups, the combination of targeted AGuIX-Bi-cRGD and 3 × 6 Gy led to a significant increase in the percentage in the infiltration of intratumoral CD3^+^ (*p* < 0.05) and CD8^+^ (*p* < 0.05) T cells, respectively (Fig. 7b and c). In line with previously findings [110, 116, 117], our results suggest that the therapeutic effect of targeted AGuIX-Bi-cRGD NPs in combination with fractionated irradiation is primarily due to the CD3^+^ CD8^+^ T cell - mediated immune response.

## Conclusion

In summary, we have successfully developed a novel integrin-binding AGuIX-Bi-cRGD theranostic nanoparticle that improves tumor targeting and enhances the antitumor efficacy of fractionated radiation therapy in lung cancer. Our findings show that RGD-tagging increases the internalization of AGuIX-Bi NPs by up to two-fold in lung cancer cell lines and in an NSCLC tumor-bearing murine model, while their ultrasmall size ensures efficient renal clearance following systemic administration. Intravenous injections of 300 mg.kg^− 1^ of AGuIX-Bi-cRGD, in combination with fractionated irradiation, led to minimal systemic toxicity, inhibition of LLC tumor growth, and prolonged median survival of treated mice. This treatment combination also shifts “cold” lung tumors to “hot” ones by inducing immunogenic cell death and enhancing the accumulation of tumor-infiltrating cytotoxic T cells. Our results indicate that AGuIX-Bi-cRGD NPs offer a promising novel approach for treating highly aggressive and immune-resistant lung cancers using radiotherapy.

## Electronic supplementary material

Below is the link to the electronic supplementary material.

Supplementary Material 1

## Data Availability

No datasets were generated or analysed during the current study.
