# Supplementary material for: Targeted-theranostic nanoparticles induce anti-tumor immune response in lung cancer
Source: J Nanobiotechnology. 2025 Jul 1;23:466. doi: 10.1186/s12951-025-03542-4 (PMC12210714; doi:10.1186/s12951-025-03542-4)
Supplement: Supplementary file 1 — Supplementary Material 1 [file 12951_2025_3542_MOESM1_ESM.docx]

**Supporting Information for**

**Targeted-theranostic nanoparticles induce anti-tumor immune response in lung cancer**

**I. Supplementary experimental procedures**

**cRGD quantification via BCA assay.** cRGD quantification was performed using a bicinchoninic acid (BCA) protein Assay (#23225, Thermo Scientific). Amino acids in biomolecules were quantified by a colorimetric assay based on the Biuret reaction. Cu^2+^ ions present in the solution were reduced to Cu^+^ ions, in an alkaline medium in the presence of proteins. Two molecules of BCA react with the reduced ion to form a violet complex with a high absorbance at 562 nm. The more intense the absorbance of the complex, the more numerous the peptide bonds. This colorimetric growth was linear, enabling a calibration curve of the peptide to be created. As per the protocol, 0.1 ml of each calibrant and the unknown sample were mixed with 2.0 ml of the BCA™ working reagent in a tube. The mixture was incubated at 37 °C for 4 h and absorbance was read at 562 nm. A calibration curve was generated for concentrations ranging from 0 to 130 µM.

***ζ-potential and dynamic light scattering (DLS) measurements.*** NPs were diluted in an aqueous solution containing 0.01 M NaCl to a concentration of 10 g. L^-1^. The size distribution and ζ-potential were measured using a Zetasizer NanoS DLS instrument (Dynamic Light Scattering, with a He-Ne laser at a wavelength of 633 nm) from Malvern Instruments.

***Relaxivity measurements.*** Relaxivity was measured on a Bruker Minispec mq60 NMR analyzer (Bruker) at 37 °C and a magnetic field strength of 1.4 Tesla (60 MHz). NPs were analyzed at 100 or 50 g. L^-1^ concentrations.

**High-performance liquid chromatography with UV detection.** NPs analysis was done using High-Performance Liquid Chromatography with UV detection (HPLC-UV). A Shimadzu Prominence series UFLC system comprised with an LC-20 AD liquid chromatograph, a CBM-20A controller bus module, a CTO-20A column oven, and an SPD-20A UV-visible detector was utilized. UV-visible absorption was monitored at 295 nm. Separation was achieved using a C4 reverse-phase HPLC column (Jupiter®, 5 µm, 300 A, 150 x 4.6 mm) at a flow rate of 1 mL. min^-1^.

The gradient elution began with a starting mobile phase composition of 95% solvent A and 5% solvent B (where A = H2O / ACN / TFA: 98.9 / 1 / 0.1% v, and B = ACN / H2O / TFA: 89.9 / 10 / 0.1% v) over a duration of 5 minutes. Subsequently, a gradient was applied, increasing solvent B from 5% to 90% over 15 minutes: this concentration of solvent B was maintained for 5 minutes before being reduced to 5% over 5 minutes to re-equilibrate the system. The system was then held at this concentration for an additional 5 minutes.

***Cellular uptake analysis using flow cytometry.*** LLC cells (5 × 10^5^ cells per well) were seeded in 6-well plates. The next day, cells were treated with 1 mg. mL^-1^ Cy5.5 labeled AGuIX-Bi and AGuIX-Bi-cRGD NPs diluted in base DMEM-GlutaMax media for 24 hours. Cells were collected and fixed with fixation buffer (#420801, BioLegend, USA) for 20 min at RT, then washed with staining buffer (#420201, BioLegend, USA), re-suspended in PBS, and analyzed using JF Fortessa HTS at the DFCI Flow Cytometry Core. Untreated cells were used as a control to determine gating parameters. FlowJo (version 10.9.0, FlowJo LLC) software was used gating and analysis of the cell population.

***Cellular receptor blocking assay.*** A549 cells (1 × 10^5^ cells per well) were seeded in 48-well plates covered with 8 mm coverslips (#101413-530, VWR, Germany), allowed to grow for 24 h, then incubated with 3.4 µg. mL^-1^ of anti- integrin β5 (#3629T, Cell Signaling Technology) and 5.6 µg. mL^-1^ of anti- integrin α5 (#4705T, Cell Signaling Technology), or 2:100 anti- avβ3 integrin (#ZMS1030-25UL, Sigma-Aldrich) antibodies for 2 hours. Cy 5.5 labeled AGuIX-Bi-cRGD NPs was added to a final concentration of 1 mg. mL^-1^ and cells were incubated for 4 hours, fixed with 10% NBF (#EK-4499-10L, Medsupply Partners) for 10 minutes, counterstained with DAPI (#D1306, Thermo Fisher Scientific) for 20 minutes and the coverslips were mounted with Fluoromount-G mounting medium (#00-4958-02, Thermo Fisher Scientific). Cells were imaged on a fluorescence microscope (Axio Observer 7, Carl Zeiss Microscopy) using a 100X oil objective.

***𝛄-H2AX assay.*** A549 (1 × 10^5^ cells per well) and LLC cells (1 × 10^5^ cells per well) were seeded in 48-well plates covered with 8 mm coverslips (#101413-530, VWR), allowed to grow for overnight, then incubated with 1 mg. mL^-1^ of AGuIX-Bi or AGuIX-Bi-cRGD for 4 hours in base RPMI-1640 (in A549 cells) or in HBSS solution (#14175095, Gibco) (in LLC cells) for 1 hour. NPs solutions were replaced with a complete growth medium, and cells were irradiated on the SARRP to 6 Gy. Thirty minutes post-treatment, cells were fixed, permeabilized with 0.3% Triton X-100 for 20 minutes, and blocked with 10% FBS for 1 hour at RT; they were then stained with anti-γH2AX-AlexaFlour 594 (#613410, BioLegends) and DAPI (#D1306, Thermo Fisher Scientific) for 2 hours and were mounted with Fluoromount-G mounting medium. Cells were imaged on a fluorescence microscope (Axio Observer 7, Carl Zeiss Microscopy) using a 100X oil objective and analyzed using an in-house ImageJ (v 1.52p) foci counter macro as previously described [1].

***In vitro cytotoxicity MTT assay.*** A549 or HUVEC cells (1 × 10⁴ per well) were plated in a 96-well plate and incubated for 24 hours before being exposed to AGuIX-Bi or AGuIX-Bi-cRGD, dispersed in either RPMI-1640 medium or HBSS solution, at varying concentrations (0.1, 0.5, 1, 5, 10, 15, and 20 mg. mL^-1^). Following incubation for 4 hours in A549 cells and 1 hour in HUVEC cells, the nanoparticle solutions were replaced with 10% of a 5 mg. mL^-1^ MTT solution (#M5655, Sigma-Aldrich) in complete growth media and incubated for another 4 hours at 37°C. The resulting formazan crystals were dissolved in 100 µL of DMSO, and absorbance was recorded at 570 nm. Relative cell viability (%) was calculated as previously described [2].

***In vitro HMGB1 expression analysis using flow cytometry.*** A549 cells (0.5 × 10^6^ cells per well) were seeded in a 12-well plate. The next day, cells were treated with 1 mg. mL^-1^ of AGuIX-Bi or AGuIX-Bi-cRGD for 4 hours in base RPMI-1640. NP solutions were replaced with complete growth medium, and cells were exposed to 6 Gy of radiation on the SARRP. Twenty-four hours post-treatment, cells were collected, fixed (#420801, BioLegend), permeabilized (#421002, BioLegend), and stained for 30 minutes with 4 µl of anti-HMGB1-PE (#651403, BioLegend) per 100 µl of cell suspension at RT, washed, and analyzed using JF Fortessa HTS at the DFCI Flow Cytometry Core on the next day. FlowJo (version 10.9.0, FlowJo LLC) software was utilized for gating and analysis of cell populations.

***In vitro HMGB1 release analysis using ELISA.*** A549 cells (0.2 × 10^6^ cells per well) were seeded in a 24-well plate. The next day, cells were treated with 1 mg. mL^-1^ of AGuIX-Bi or AGuIX-Bi-cRGD for 4 hours in base RPMI-1640. NP solutions were replaced with complete growth medium, and cells were exposed to 6 Gy of radiation on the SARRP. Ninety-six hours post-treatment, supernatants were collected and analyzed with Human HMGB-1 ELISA Kit (#EEL047, Thermo Fisher Scientific) according to the manufacturer’s protocol.

**II. Supplementary figures**

**Fig S1** SARRP treatment plan for a mouse receiving 6 Gy radiation. A cone beam computed tomography image was obtained at 60 kVp with 0.8 mA current and a 1 mm Al filter to locate the tumor's isocenter in MuriSlice. Subsequently, a single posterior-anterior beam at 220 kVp with 13 mA current and a 0.15 mm Cu filter was used to deliver a dose of 6 Gy to a 10 x 10 mm^2^ collimated field.

**Fig S2** AGuIX-Bi-cRGD in combination with radiation therapy enhanced antitumor efficacy *in vivo*. SARRP CT images of the mouse bearing subcutaneous LLC tumor exposed to AGuIX-Bi-cRGD and RT at day 1 and 23 of the treatment, red arrow indicates the tumor location.

**Fig S3** Flow cytometry analysis strategy for cellular internalization of Cy 5.5-labeled AGuIX-Bi and AGuIX-Bi-cRGD nanoparticles. **a** Histograms showing the gating strategy for LLC cells negative (left) and positive (right) for GFP and **b** for Cy 5.5 negative (left) and positive (right) after exposing to Cy 5.5-labeled AGuIX-Bi and AGuIX-Bi-cRGD nanoparticles. The threshold for gating between positive and negative fluorescence populations were established from non-treated negative control.

**Fig S4** Representative images of A549 cells treated for 2 hours with anti- αv and β5 integrin antibodies, prior to exposure to Cy 5.5-labeled AGuIX-Bi-cRGD NPs. Scale bar: 20 µm

**Fig S5** MTT assay with different concentrations of AGuIX-Bi-cRGD nanoparticles in HUVEC endothelial cell line.

**Fig S6** Specific biodistribution by ICP-MS gadolinium monitoring of AGuIX-Bi compared to AGuIX-Bi-cRGD in **a** kidney, **b** liver, **c** spleen, **d** blood, **e** lung and **f** heart. **g** Graph illustrating the confidence interval, expressed in percentage plus or minus, for obtaining the theoretical initial molar ratio of 70% bismuth / 30% gadolinium of AGuIX-BI as a function of organs and various time points (n= 4-8 in each group. Graphs show mean ± SD).

**Fig S7** *In vivo* blood toxicology evaluation of AGuIX-Bi and AGuIX-Bi-cRGD in combination with radiation therapy **a** Eosinophil (EO) **b** Basophil (BA) **c** Hematocrit (HCT) **d** Hemoglobin concentration (Hb) **e** Mean corpuscular volume (MCV) **f** Red cell distribution width (RDW) **g** Platelet crit (PCT) **h** Mean platelet volume (MPV) and **i** Platelet distribution width (PDW) (n= 3 in each group. **p* < 0.05, ns= not significant, two-tailed unpaired Student’s t-test. Graphs show mean ± SD).

**Fig S8** Flow cytometry gating strategy for HMGB1 expression in A549 cells exposed to 1 mg. ml^-1^ of AGuIX-Bi and AGuIX-Bi-cRGD nanoparticles alone or in combination with 6 Gy showing negative (left) and positive (right) populations. The threshold between positive and negative fluorescence populations were identified from non-treated negative control.

**Fig S9** Assessment of HMGB1 release by ELISA in A549 cells 96 hours post-treatment with 6 Gy alone or in combination with AGuIX-Bi or AGuIX-Bi-cRGD nanoparticles (n= 4 in each group. **p* < 0.05, ns = not significant, two-tailed unpaired Student’s t-test. Graphs show mean ± SD).

**III. Supplementary references**

1. Brown N, Rocchi P, Carmès L, Guthier R, Iyer M, Seban L, et al. Tuning ultrasmall theranostic nanoparticles for MRI contrast and radiation dose amplification. Theranostics. 2023;13.

2. Satyavani K, Gurudeeban S, Ramanathan T, Balasubramanian T. Toxicity study of silver nanoparticles synthesized from Suaeda monoica on Hep-2 cell line. Avicenna J Med Biotechnol. 2012;4.
